# Irys Extract

**DOI:** 10.1093/bioinformatics/btx437

**Published:** 2017-07-11

**Authors:** Rani Arielly, Yuval Ebenstein

**Affiliations:** School of Chemistry, Raymond and Beverly Sackler Faculty of Exact Sciences, Center for Nanoscience and Nanotechnology, Tel Aviv University, Tel Aviv, Israel

## Abstract

**Summary:**

Irys Extract is a software tool for generating genomic information from data collected by the BioNano Genomics Irys platform. The tool allows the user easy access to the raw data in the form of cropped images and genetically aligned intensity profiles. The latter are also made compatible with the BED format for using with popular genomic browsers such as the UCSC Genome Browser.

**Availability and implementation:**

Irys Extract has been developed in Matlab R2015a, it was tested to work with IrysView 2.4.0.15879 and AutoDetect 2.1.4.9159, and it currently runs under Microsoft Windows operating systems (7-10). Irys Extract can be downloaded alongside its manual and a demo dataset at http://www.nanobiophotonix.com and https://sites.google.com/site/raniarielly/.

**Supplementary information:**

Supplementary data are available at *Bioinformatics* online.

## 1 Introduction

Single-molecule techniques for genetic analysis are revolutionizing our understanding on cellular regulation. The Irys instrument from BioNano Genomics Inc. (http://bionanogenomics.com) is designed to stretch long chromosome segments inside an array of nanochannels for genetic analysis. Applying sequence specific fluorescent labeling of the DNA and imaging the stretched molecules, results in an optical map for each DNA molecule reflecting the underlying sequence (Das *et al.*, 2010; Lam *et al.*, 2012). This technology is geared toward analysis of DNA ranging in size between 10 kbp and 1 Mbp (Austin *et al.*, 2011), hence providing access to information on the individual gene length scale, with throughput of 200x coverage in about 24 hours (Pang *et al.*, 2017).

Besides providing the ability to acquire images of stretched and labeled DNA molecules, BioNano Genomics provides sophisticated software tools (AutoDetect and IrysView) that perform the image processing automatically in order to digitize the optical maps and allow the user to perform tasks like de novo assembly, alignment and variation detection (Amini *et al.*, 2014). Despite not being supported by the company, the Irys is able to image in two color channels, opening opportunities to study information beyond genetics. Our lab has already demonstrated fluorescence labeling of epigenetic marks (Michaeli *et al.*, 2013; Nifker *et al.*, 2015; Shahal *et al.*, 2016), DNA damage lesions (Zirkin *et al.*, 2014) and genetic fingerprints of short bacteriophage genomes (Grunwald *et al.*, 2015), all requiring intensity based analysis not available via the standard IrysView platform. In addition, comfortable access to a raw form of the data such as molecule images and intensity profiles may reduce false positives/negatives when using custom enzymes or dyes.

The purpose of Irys Extract is to mediate the capabilities of the Irys platform and the requirement of the advanced user by providing molecule images and genome aligned intensity profiles based on the automated analysis of the Irys platform.

Irys Extract also introduces features such as controllable molecule image margin sizes and optical profiles summation range, an advanced background subtraction scheme as well as controlling the genome fitting process. In addition, Irys Extract supports two color optical maps and allows filtering the molecules according to: features on the second channel, confidence score, molecule length, genome mapping location, molecule IDs and amount of deviation from the mapped region. The program performs profile averaging and can output seamlessly stitched fields of view for publication purposes. Creation of a local database allows discarding the original data and thus minimizing the storage needs and overall data traffic in the lab ∼1000 fold. All of these features are controllable *via* a friendly graphical user interface (GUI).

## 2 Methods and features

**Fig. 1:**
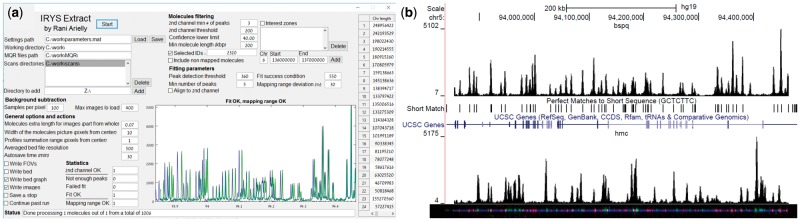
The graphical user interface (GUI) of Irys Extract and its typical output. (**a**) The GUI is divided into several areas: directories, molecule filtering, fitting parameters, background subtraction, general options and actions, statistics, chromosomes lengths, status bar and intensity graph. The specific function of each item is described in the online manual (please see the Availability and Implementation section). (**b**) Bottom: Image of a molecule dyed with YOYO-1 (blue) and marked with sequence specific TAMRA dye labelling using the nicking enzyme Nt.BspQI for the GCTCTTC recognition sequence (red fluorescence spots), and with Dibenzylcyclooctyne-Sulfo-Cy5 fluorescent labeling of Azide-tagged 5-Hydroxymethylcytosine (green fluorescence spots). Top: screen capture from the University of California at Santa Cruz Genome Browser (Kent *et al.*, 2002) (http://genome.ucsc.edu). The screenshot contains intensity profiles for the genetic red (top) and epigenetic green (bottom) channels in the molecule image below. The green signal was stretched according to the Irys data in order to best fit the data of the red channel to the known locations of the GCTCTTC sequence in the genome assembly hg19, Feb 2009 (shown as a track of black tags in the middle of the screen capture). Local genes are indicated in the blue UCSC gene track, showing the advantage of this single molecule method for correlation with published genomic features in terms of required length scale of the data. The top of the image shows the chromosome and position to which the molecule was mapped onto

Irys Extract appears as a single window which is shown in Figure 1a. The GUI is divided into several areas. The specific function of each item is described in the online manual (please see the Availability and Implementation section). The user enters his desired values in each field in the GUI or loads them from a previously saved settings file, and initiates the program. Irys Extract starts by constructing a database composed of data aggregated from the standard Molecule quality report files created by the IrysView program for a single run or a merged set of runs. The program adds to the database the relevant information regarding the raw image locations from the AutoDetect folders of the runs and filters it according to intensity requirements from the channels, confidence score, length of the molecules and specific genome locations or specific molecular IDs from IrysView. For each molecule on this filtered database the program stitches the relevant fields of view images for all of the color channels, and crops the resulting image according to the settings in the GUI (see Supplementary Information). The program also subtracts the image background by applying a method of dividing a field of view image into 64 regions, and calculating a local background for each region by averaging a number of image frames (controlled in the GUI) while searching and excluding any signal that is not typical for the background (see Supplementary Information). Besides saving this final cropped image, the program also saves the averaged background image of each scan file, and the cropped versions of the raw and background images for future, faster use of the program and for convenient local data archiving due to the decreased volume of the cropped data. Irys Extract will then calculate intensity profiles, and use the genetic marker channel for refining the genetic fitting by passing the profile through a peak detection function (http://www.billauer.co.il/peakdet.html) and aligning the peaks with the reference marks proposed by the Irys system. The fitting result of the genetic marker will show on the GUI graph. The success rate of this fitting procedure was tested to be ∼99.98% at a requirement of ∼1 pixel accuracy. The program will then save the aligned intensity profiles in the ‘bedgraph’ format (Hung and Weng, 2016) in several forms: separately for each molecule, genetically tiled separately to the minimal number of files, and averaged molecular intensity profiles in a single file. In each form different color channels will be saved in separate files.

These intensity profiles can then be loaded into a genome browser like the one from the University of California at Santa Cruz (Kent *et al.*, 2002) (http://genome.ucsc.edu), in order to examine their features against those of the tracks that are available in the browser such as genes and regulatory elements. An example is shown in Figure 1b together with an image of the molecule from which the intensity profiles were produced. Using Irys Extract we were able to map epigenetic marks to the reference genome and perform various genome wide and locus specific bioinformatics analysis for this data using standard software tools.

## Funding

This work has been supported by the BeyondSeq consortium [EC program 634890], the European Research Councils starter grant [337830] and the Tel Aviv University Center for Nanoscience and Nanotechnology.

*Conflict of Interest*: none declared.

## Supplementary Material

Supplementary Figures
