# Supplementary material for: Irys Extract
Source: Bioinformatics. 2017 Jul 11;34(1):134–6. doi: 10.1093/bioinformatics/btx437 (PMC5870776; doi:10.1093/bioinformatics/btx437)
Supplement: Supplementary Figures [file supplementary_info_btx437.docx]

Irys Extract – Supplementary Information

Rani Arielly and Yuval Ebenstein

*School of Chemistry, Raymond and Beverly Sackler Faculty of Exact Sciences, Tel Aviv University, 69978 Tel Aviv, Israel*

Image Stitching and Background Subtraction

For each molecule, based on the data provided by the Irys system, Irys Extract produces a single, cropped, high quality image by performing a series of action. The program first resizes the images depending on their color to account for the differences in optics in the different color channels. Next, it rotates and stitches the relevant fields of view images for all of the color channels, and crops the resulting single image according to the image margins set in the GUI. An example for this stitching and cropping process is presented in Figure S1.


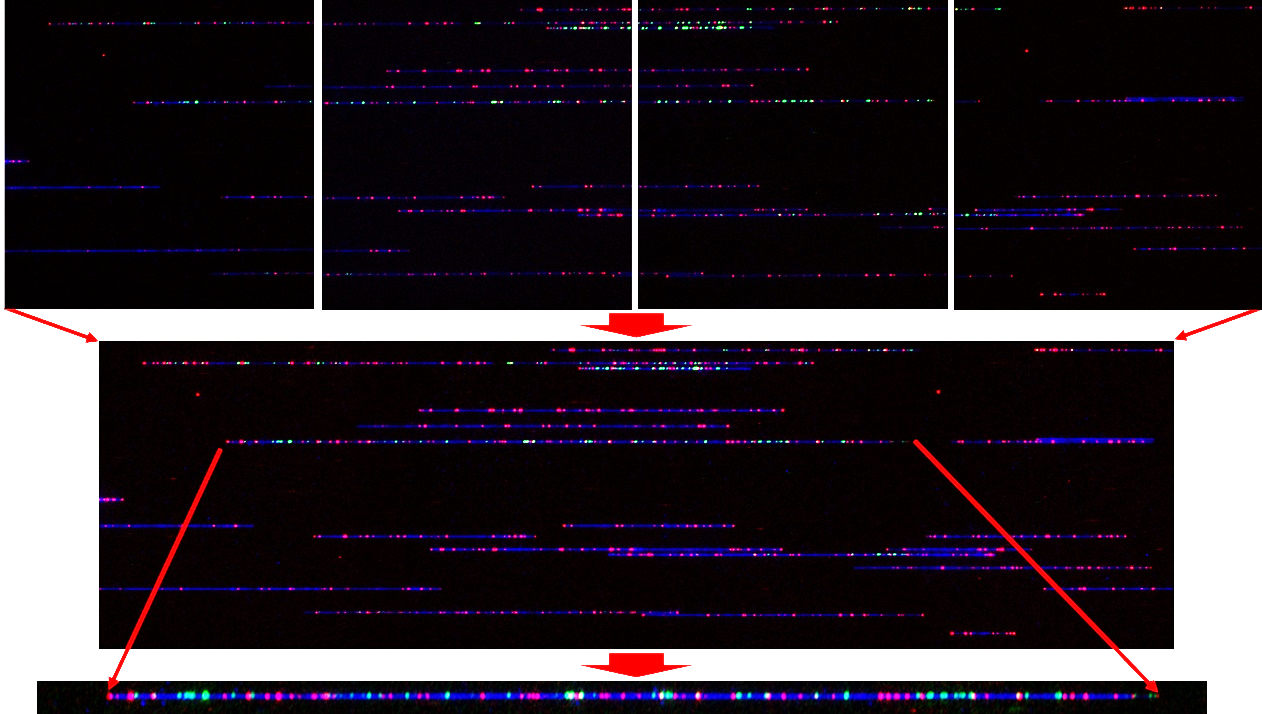


Figure S1. Stitching and cropping the molecule. These images contain a molecule that was mapped to a location of interest in the genome. Parts of the molecule are seen in 4 consecutive fields of view. Based on the Irys data, these fields of views are stitched together and only the pixels around the molecule's location are saved.

The program also subtracts the image background by applying a method of dividing the field of view into 64 segments, and for each – averaging a number of images (controlled in the GUI) while searching and excluding any signal that is not typical for the background signal level. This procedure results in a quantitative fluorescence signal throughout the field view. Example of such background subtraction and the resulting molecule images can be seen in Figure S2.


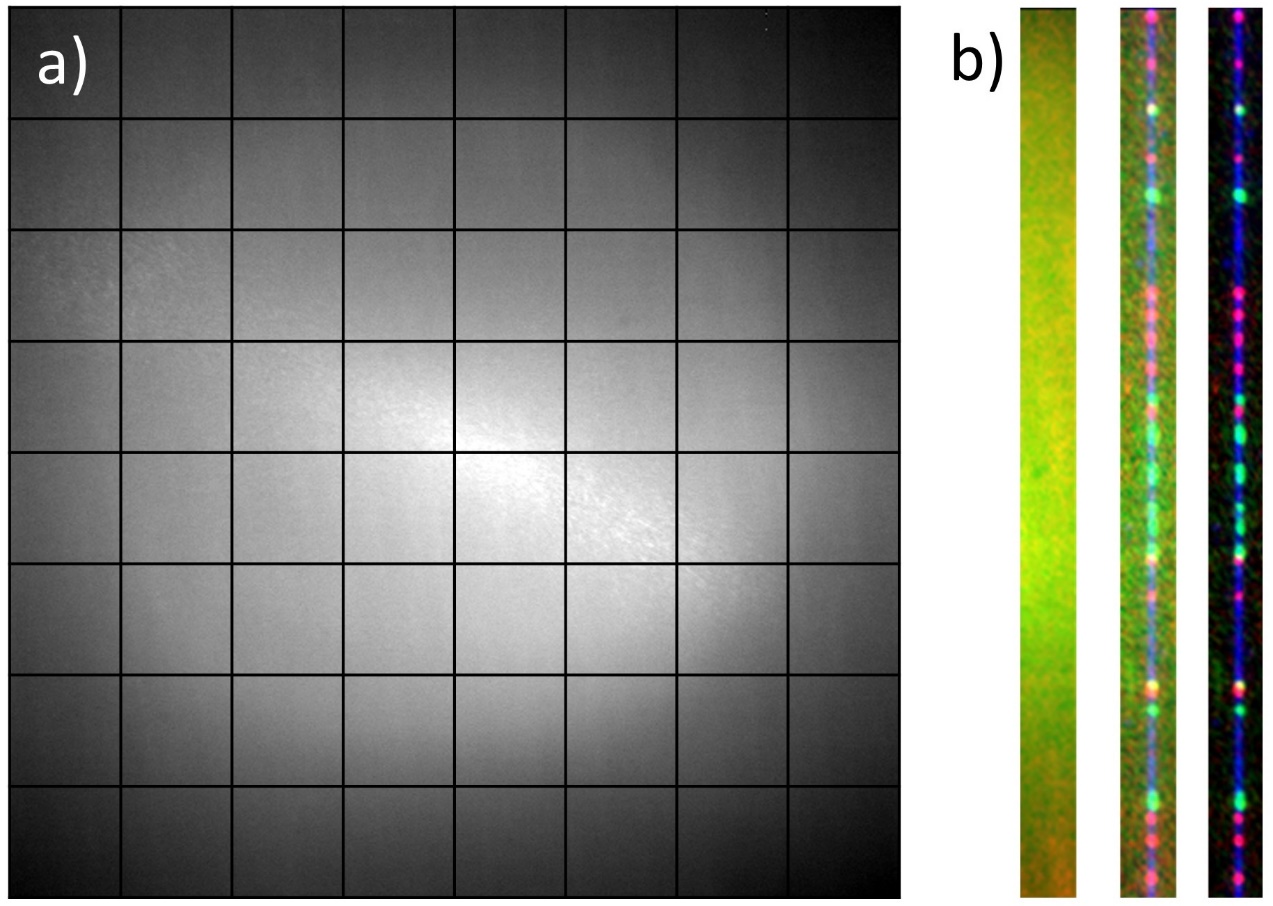


Figure S2. The background subtraction feature. a) Non uniform background signal was created by non-uniform illumination. This is remedied by averaging a set number of images (controlled in the GUI), while discarding data above a certain threshold which is unique to that pixel's region in the image. The image is divided into 64 regions. b) The resulting cropped molecule image (inside one field of view) before (middle) and after (right) the background subtraction, and the local background image (left). One can see that the features on the molecule are much clearer after the background subtraction.
